# N400 ERPs for actions: building meaning in context

**DOI:** 10.3389/fnhum.2013.00057

**Published:** 2013-03-04

**Authors:** Lucía Amoruso, Carlos Gelormini, Francisco Aboitiz, Miguel Alvarez González, Facundo Manes, Juan F. Cardona, Agustín Ibanez

**Affiliations:** ^1^Laboratory of Experimental Psychology and Neuroscience, Institute of Cognitive Neurology, Favaloro UniversityBuenos Aires, Argentina; ^2^Institute of Neuroscience, Favaloro UniversityBuenos Aires, Argentina; ^3^National Scientific and Technical Research CouncilBuenos Aires, Argentina; ^4^Department of Psychiatry, Medical School, Interdisciplinary Center of Neuroscience, Pontificia Universidad CatólicaSantiago, Chile; ^5^Institute of Neurology and Neurosurgery, Universidad de la HabanaCiudad de La Habana, Cuba; ^6^Laboratory of Cognitive and Social Neuroscience, Universidad Diego PortalesSantiago, Chile

**Keywords:** N400, action comprehension, action meaning, language, contextual integration, fronto-temporo-parietal network

## Abstract

Converging neuroscientific evidence suggests the existence of close links between language and sensorimotor cognition. Accordingly, during the comprehension of meaningful actions, our brain would recruit semantic-related operations similar to those associated with the processing of language information. Consistent with this view, electrophysiological findings show that the N400 component, traditionally linked to the semantic processing of linguistic material, can also be elicited by action-related material. This review outlines recent data from N400 studies that examine the understanding of action events. We focus on three specific domains, including everyday action comprehension, co-speech gesture integration, and the semantics involved in motor planning and execution. Based on the reviewed findings, we suggest that both negativities (the N400 and the action-N400) reflect a common neurocognitive mechanism involved in the construction of meaning through the expectancies created by previous experiences and current contextual information. To shed light on how this process is instantiated in the brain, a testable contextual fronto-temporo-parietal model is proposed.

## Introduction

Comprehension of everyday actions is a key component of human cognition. As social animals, we constantly move in an environment where we actively perceive others' movements as a form of meaningful behavior (Blakemore and Decety, 2001; Gallese et al., 2007; Fitch et al., 2010). In other words, we perceive body movements as the expression of peoples' intentions and beliefs and as cues as to how we might respond or interact with them. Accordingly, comprehension can be considered as a cognitive process that uses verbal and non-verbal resources in order to build up meaning as a coherent and unified depiction of a given situation. Thus, gestures, gaze, body postures, and goal-directed motor behaviors are a powerful source of communication that enables us to accurately interact with our conspecifics in daily life by disambiguating speech, identifying emotional states and understanding other peoples' aims.

In addition, the semantic significance of an action event is context-embedded; this means that the observation and interpretation of the behavior of others is not only intentional and interactional, but also highly context-dependent (Wurm et al., 2012). Objects, persons, and the relationships amongst them are not perceived as detached from a social background; rather, they are perceived as a whole meaningful act in which online verbal and non-verbal information and previous knowledge about similar situations are integrated by the brain in a flowing manner. Based on this integration, context helps us interpret events by building up expectations about what is more likely to happen in a given situation (Bar, 2004, 2009; Ibanez and Manes, 2012). Similarly, compatible contextual settings would constrain expectations in a facilitatory fashion, whereas incompatible ones would cause interference and would demand an extra cognitive effort to disentangle the meaning of that particular situation (Wurm and Schubotz, 2012).

Over the last few decades, event-related potentials (ERPs) have been used to investigate how meaning is processed in the brain and how contextual information affects this processing (Ibanez et al., 2012b). A specific component, the N400 (a negative-going voltage occurring approximately 400 ms after a meaningful stimulus onset), has been linked to the semantic integration of a given stimulus into a previous context. Although this component was first discovered in response to semantic anomalous sentence endings in linguistic paradigms (Kutas and Hillyard, 1980), similar effects have been recently observed for non-linguistic material involving meaningful actions (e.g., Sitnikova et al., 2003).

In the linguistic domain, the N400 is a robust electrophysiological marker of semantic processing. While its latency remains relatively constant (Kutas and Federmeier, 2011), the N400 amplitude has been shown to be sensitive not only to the degree of semantic incongruity *per se* but also to several other factors. For example, classical studies have suggested that low-frequency words elicit larger amplitudes than high-frequency ones (Van Petten and Kutas, 1990). The N400 amplitude is also reduced by repetition, such that a word that has recently appeared exhibits a less negative response when it is repeated than when it is not (Rugg, 1985). Expectancy or cloze-probability also modulates the N400 response (Kutas and Hillyard, 1984), with less expected sentence endings showing larger N400 responses than highly expected ones, even when both endings are semantically congruent. Further, its amplitude is also affected by priming because unrelated items show larger N400 amplitudes relative to related items (Bentin et al., 1985). In addition, word-like letter strings (or pseudo-words) have also been shown to enhance N400 amplitudes when compared with words (Rugg and Nagy, 1987). Finally, another reported effect is the N400-concreteness effect. This effect is typically observed in relation to the processing of concrete and abstract nouns, with concrete nouns eliciting enhanced frontal N400 responses compared to abstract nouns (Kounios and Holcomb, 1994).

However, some of these factors are not restricted to linguistic material, and similar effects have also been observed in response to action-related stimuli. For example, pseudo-actions have been shown to modulate N400 amplitudes in a similar manner to pseudo-words (Proverbio and Riva, 2009). Repetition and concreteness (Van Elk et al., 2008, 2010a) as well as expectancy (Reid and Striano, 2008) in non-verbal paradigms also lead to analog modulations as those observed for verbal items.

Furthermore, action-elicited N400 waves have been shown to resemble the shape and timing of linguistic N400 waves, suggesting a functional similarity between both negativities. Nevertheless, most of the previous studies have also reported some differences. For example, while the N400 elicited by linguistic material has a maximum peak over the central and parietal regions, the N400 observed for actions seems to be more frontally distributed. In addition, some studies have also reported an early latency during the processing of action-related material, perhaps driven by the pictorial characteristics of the stimulus being processed (Holcomb and McPherson, 1994; McPherson and Holcomb, 1999; Hamm et al., 2002). Together, these differences lead to questions regarding the neural architecture necessary to build up meaning across modalities and the temporal aspects involved in this complex process. Extensive behavioral, lesion, and functional imaging literature suggest that “meaning” is an emergent process which takes place in a widely distributed neural network, simultaneously open to verbal and non-verbal stimuli and that “comprehension” is a predictive, flexible, and context-dependent process indexed by a wide distributed brain activity (Federmeier and Laszlo, 2009; Kutas and Federmeier, 2011).

Although most current positions share this distributed view, there is still no full agreement on how to interpret N400 extant data, and different explanations have been proposed. For example, it has been recently posited that this component would reflect a semantic unification process instantiated by a network comprising of storage (middle/superior temporal gyrus, MTG/STG), multimodal (inferior frontal gyrus, IFG) and control retrieval areas (dorsolateral prefrontal cortex, DLPFC), with a contribution of parietal areas (e.g., angular gyrus, AG) in giving support to this unification (Baggio and Hagoort, 2011) through sensorimotor integration-related processes. Similarly, another interesting proposal suggests that the N400, as an index of semantic facilitation, would originates in a network where lexical representations are stored in temporal regions (inferior temporal cortex, MTG and superior temporal sulcus, STS) and is accessed by integrative areas (anterior temporal lobe and AG) which together would incorporate the incoming inputs into the semantic context that is being built (Lau et al., 2008). In this model, the IFG would control the top–down lexical semantic retrieval and mediate the selection among candidate representations. Finally, an alternative approach (Federmeier and Laszlo, 2009) suggests that the N400 reflects a temporal binding process that “glues” spatially distributed information into a synchronic and unified activity experienced as the meaning of the stimulus being processed. The medial temporal lobe, based on its strategic localization and connections, would be a key area in mediating such binding.

Together, despite their differences, these interpretations point to a constructive and context-dependent view of meaning supported by a common distributed semantic network comprising unimodal, multimodal, and storage areas. However, the aforementioned accounts have been mainly proposed for the classical N400 effect elicited by words and to our knowledge, no current particular model has been proposed to interpret the N400 effect elicited by meaningful actions.

Moreover, an important step in the development of an action-N400 model is to assess how the brain would anticipate and integrate contextual information in order to have access to action-meaning. Current models of conceptual representations (Kiefer and Pulvermuller, 2012) provide an alternative. These models propose distributed and modality-specific sensory and action representations, based on a bidirectional coupling between motor and language areas. Similarly, current theories of abstract conceptual representations indexed by the anterior temporal lobe, as well as the brain predictive coding account also provide explanatory heuristics that would be integrated into an N400 account. However, no previous work has assessed whether these theories are well situated as explanatory models of the N400 for actions.

Thus, we have selectively focused on the recent findings from action comprehension studies that have used the N400 as an electrophysiological measure of semantic contextual integration. For instance, our review spotlights on action language paradigms which are focused on N400. By doing so, we hope to delineate a specific characterization of the N400 component, propose a fronto-temporo-parietal testable model which integrates the action-related data to current knowledge about the classical N400, and encourage a discussion as to what the N400 indexes.

**Figure 1:**
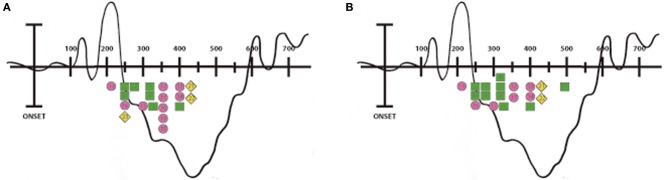
**Peak latencies of the N400 ERPs.** Illustration showing the timing of the N400 ERPs reported in the reviewed studies. Each number corresponds to a study (please see Table 1 for information regarding the enumeration). Everyday action studies are indicated with a green square, speech and co-speech gestures studies with a pink circle and current motor events studies with a yellow diamond. Picture **(A)** corresponds to the N400 peaks reported in the left hemisphere and picture **(B)** corresponds to those reported in the right hemisphere.

We have structured this review according to three possible scenarios in which the interaction between language and action can be observed. First, we review the N400 studies based on the comprehension of daily actions. Here, the assertion is that non-verbal cues about action events are processed by the brain in the same way as verbal cues. This hypothesis implies that the construction is based on a multimodal integration process. Second, we look at N400 studies on the coupling between speech and gestures. In this domain, the link is supported by the integration of actions and words during meaning comprehension; in addition, information conveyed by both types of stimuli is processed by the brain in a qualitatively similar fashion. Third, we analyze studies concerning the influence of semantics in motor planning and execution. In these studies, action-language cooperation is supported by the bidirectional impact of sensorimotor systems and language during the preparation and execution of actions intertwined with semantic stimuli. The reviewed studies and their main findings are presented in Table 1 and Figure 1.

**Table 1:** **A summary of the reviewed studies on N400 for action comprehension**.

**Study**	**Stimuli**	**Distribution**	**Lateralization**	**Other effects**
**COMPREHENSION OF EVERYDAY ACTIONS**				
1. Sitnikova et al. (2003)	Videos	Frontal and Central	Both	N300/LPC
2. Balconi and Caldiroli (2011)	Videos	Frontal and Central	Both	
3. Reid and Striano (2008)	Videos	Frontal	Right	
4. Sitnikova et al. (2008)	Videos	Frontal and Central	Right	N300/LPC
5. West and Holcomb (2002)	Pictures (Drawings)	Frontal and Central	Right	N300
6. Mudrik et al. (2010)	Pictures (Photos)	Frontal and Central	Both	N300
7. Shibata et al. (2009)	Pictures (Photos)	Parietal	Both	N300/N800
8. Bach et al. (2009)	Pictures (Photos)	Central	Both	LPC
9. Proverbio and Riva (2009)	Pictures (Photos)	Frontal	Both	N250
10. Proverbio et al. (2010)	Pictures (Photos)	Frontal	Both	N2/RP/N230
**SPEECH AND CO-SPEECH GESTURES**				
11. Kelly et al. (2004)	Videos/Auditory Utterances	Frontal	Both	P1-N1/P2
12. Kelly et al. (2007)	Videos/Auditory Utterances	Frontal and Central	Both	
13. Kelly et al. (2010)	Videos/Auditory Utterances	Central and Parietal	Both	P2
14. Wu and Coulson (2005)	Videos/Auditory Utterances	Frontal and Central	Both	LPC
15. Holle and Gunter (2007)	Videos/Words	Broadly	Both	
16. Ozyurek et al. (2007)	Videos/Auditory Utterances	Frontal	Both	N1-P2/N300
17. Lim et al. (2009)	Videos/Words	Central and Parietal	Both	
18. Cornejo et al. (2009)	Videos/Auditory Utterances	Frontal	Left	LPC
19. Ibanez et al. (2011a,b)	Videos/Auditory Utterances	Frontal	Left	LPC
20. Ibanez et al. (2010)	Videos/Auditory Utterances	Frontal	Left	LPC
**CURRENT MOTOR EVENTS**				
21. Van Elk et al. (2008)	Words/Motor Task	Frontal and Central	Both	
22. Aravena et al. (2010)	Auditory Utterances/Motor Task	Central	Both	MP/RAP
23. Ibanez et al. (2012b)	Auditory Utterances/Motor Task	Frontal and Central	Left	MP

## The comprehension of everyday actions

Although traditionally studied in isolation as separate modules (Collins and Loftus, 1975; Fodor, 1983; Masson and Borowsky, 1998), language and sensorimotor processes seem to be integrated during the comprehension of everyday actions. Nevertheless, how this is accomplished by the brain remains unclear.

**Figure 2:**
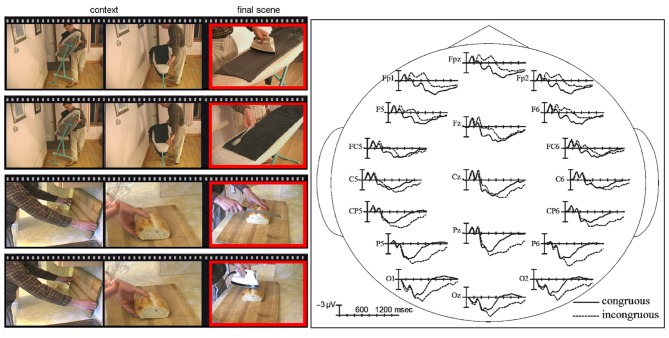
**The examples of everyday actions stimuli and N400 ERPs.** On the left side of the figure, frames taken from movie clips are shown. The first two illustrate context and the third one illustrates the congruous (e.g., a man uses an electric iron to press wrinkles from his pants) or the incongruous (e.g., a man uses a fork to iron his pants) final ending. On the right side of the figure, the waveforms of the ERPs time-locked to the incongruous final movie scenes are compared to ERPs time-locked to congruous final scenes at representative electrode sites. The data were taken from Sitnikova et al. (2003, 2008).

Recently, several electrophysiological studies based on the N400 component have provided evidence toward common functional substrates for verbal and non-verbal integration during the semantic processing of everyday actions. A more ecological approach to the study of action comprehension can be achieved using videos of dynamic events (Cornejo et al., 2009; Ibanez et al., 2011a,b). Videos elicit experiences similar to the perception of real world situations, and they can be used to obtain ERPs in an accurate fashion. In these cases, the stroke (e.g., the phase of a body movement that conveys an important dimension of a gesture meaning) can be marked precisely with a specific video frame, allowing the analysis of a dynamic event by means of a well-defined static reference point. For example, Sitnikova et al. (2003) carried out a study using short videos of people engaged in common activities (Figure 2). These actions could be performed either with the correct object (e.g., shaving with a razor) or with a wrong one (e.g., shaving with a broom). The incongruent condition elicited an N400 effect over fronto-central sites followed by a late positivity (LPC) during the 600–900 ms window. In a more recent study using videos about actions with semantic anomalous endings (e.g., combing hair with a toothbrush) similar modulations in frontal sites were found, confirming a partial overlap between the linguistic and non-linguistic domain in semantic comprehension (Balconi and Caldiroli, 2011).

Further evidence obtained by using videos has shown that unanticipated action endings (e.g., a spoon with or without food placed in the mouth at the end of a video clip) elicit a frontal N400 response (Reid and Striano, 2008) that is more pronounced over the right hemisphere and has a slight delay in its latency (peaking approximately 600 ms after stimulus onset).

Taken together, these studies suggest that the N400 effect for dynamic visual images is more frontally distributed compared to the classic N400 distribution elicited by words. Some authors have argued that this topographical difference may reflect the overlap with an earlier and anterior component: the N300 (Holcomb and McPherson, 1994; McPherson and Holcomb, 1999; Hamm et al., 2002). Typically reported in studies using pictorial stimuli, the N300 is thought to reflect object identification (Doniger et al., 2000; Schendan and Kutas, 2002, 2003; Ganis and Kutas, 2003; Folstein et al., 2008) and/or semantic processes specific to pictorial/non-verbal representations (Barrett and Rugg, 1990; Holcomb and McPherson, 1994; McPherson and Holcomb, 1999). For example, in a follow-up study, Sitnikova et al. (2008) replicated previous findings of a frontal N400 followed by a LPC (Sitnikova et al., 2003), but they also found an anterior N300 incongruity effect (starting at 250 ms after stimulus onset). According to the authors, this was possibly due to the introduction of a “cut” in the videos between the context and the final target movie scene that improved the accuracy of ERP time-locking and contribution to the N300 recording.

West and Holcomb (2002) found a similar N300/N400 complex for pictures depicting action-related stories with incongruent endings. During the earlier epoch, ERPs were focused over the right fronto-central regions (with the N300 peaking at approximately 325 ms). In the later epoch, the N400 effect (peaking at approximately 500 ms) had a more widespread distribution and was still focused in the fronto-central regions. In line with this study, Mudrik et al. (2010) reported that incongruent pictures about common actions (e.g., a man drinking from a can or potato) elicit an early fronto-central negativity starting approximately 270 ms post-stimulus onset, lasting for 330 ms and resembling the N300/N400 effect previously observed by West and Holcomb (2002) and Sitnikova et al. (2008).

Further data have shown that the inappropriate exchange of objects between two people also leads to N400 effects, suggesting that observers use salient information about hand posture and object position to interpret cooperativeness of interpersonal actions (Shibata et al., 2009).

Functional inappropriateness of the tool used in a given action (e.g., a picture of a hand holding a credit card after the presentation of a picture of a slot for coins) also leads to a right-lateralized N400 (Bach et al., 2009). Furthermore, pseudo-actions (e.g., a business woman balancing on one foot in the desert) have been reported as eliciting a frontally distributed N400 (N420) when compared with possible actions (Proverbio and Riva, 2009). Additionally, an enhanced posterior “recognition potential” (N250) was reported in this study for meaningful actions. According to the authors, these findings suggest that actions are semantically processed in early and later stages in a similar manner to linguistic stimuli. In a subsequent study, Proverbio et al. (2010) replicated these results and further showed that the N400 for actions could be modulated by gender, with larger amplitudes for women compared to men.

Taken together, the reviewed evidence suggests that daily actions elicit a more frontally distributed N400 with a bias, in some cases, toward the right hemisphere (West and Holcomb, 2002; Reid and Striano, 2008). Interestingly, negative activity seems to begin earlier at frontal sites (approximately 300 ms after stimulus onset), maybe due to the pictorial characteristics of the stimulus being processed (West and Holcomb, 2002; Sitnikova et al., 2003, 2008; Mudrik et al., 2010). Together, these findings point to a multimodal dimension of semantic understanding in which verbal and non-verbal stimuli are processed by the brain in a similar fashion.

## N400 studies on the semantic integration of speech and co-speech gestures

Another domain where the semantic integration of action and language has been studied is the one offered by the interplay of speech and gestures. Co-speech gestures are natural, spontaneous hand movements that we make while we speak. These manual actions are almost never performed in the absence of a language communicative context, suggesting that they do not have an intrinsic meaning outside of this setting. Moreover, gestures are present in social communicative situations from early childhood, suggesting that linguistic skills are later built on the platform of prelinguistic communication provided by these intentional movements (Tomasello et al., 2007).

Recent electrophysiological research on this domain supports the existence of an integrated system in which gestures and speech overlap at a semantic level. For example, Kelly et al. (2004) conducted a study in which subjects watched audiovisual segments of an actor uttering speech tokens about the salient property of an object. Utterances could be followed by a matching gesture (e.g., saying “tall” while gesturing about the “tallness” of a “tall” glass), a complementary gesture (e.g., saying “tall” but gesturing to the “thinness” of the “tall” and “thin” glass), a mismatching gesture (e.g., saying “tall” while gesturing about the “shortness”) or no gesture at all (baseline). The main finding was the mismatched condition elicited a right-lateralized N400 compared to the matched condition. In addition, early pre-semantic components (P1-N1 and P2) were observed in the bilateral occipital and frontal regions. The P1–N1 was more positive for the complementary condition relative to the other gestures, except the mismatching one. According to the authors, these results suggest that gestures are integrated with speech at the early and late stages of language processing. In a follow-up study, Kelly et al. (2007) replicated the fronto-central N400 effect that was previously found for incongruent conditions. The authors also showed that the semantic processing of gesture information is not entirely automatic. In addition, under some circumstances (e.g., when explicit instructions about whether to integrate gestures and speech are given), this semantic processing is likely to be under a certain degree of cognitive control (Kelly et al., 2010).

**Figure 3:**
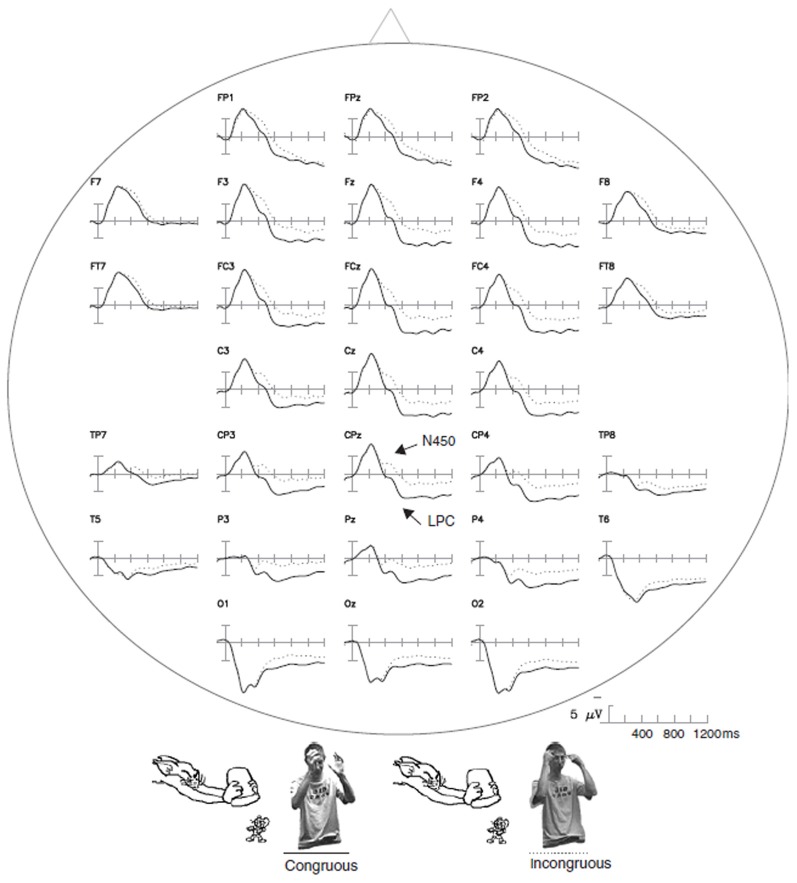
**The ERP waveforms for gestures embedded in complex contexts.** The ERP responses time-locked to the onset of congruous and incongruous gestures paired with video segments of cartoons are shown. These data were taken from Wu and Coulson (2005). The arrows indicate congruency effects indexed by N450 and LPC modulations.

Gestures embedded in a more complex context have also elicited an N400 effect (Wu and Coulson, 2005). Cartoon segments were presented along with videos of an actor performing pantomimes that could either match the preceding cartoon or not. Incongruous gestures were found to elicit a negative component peaking at approximately 450 ms, largest over fronto-central sites, followed by a LPC for congruous items peaking at 740 ms (Figure 3). According to the authors, this late positivity would reflect decision-related brain activity (e.g., evaluation and categorization of the stimuli).

Further empirical evidence was provided by Holle and Gunter (2007). Sentences that contained an ambiguous word were accompanied by a disambiguating gesture hinting at one of the two possible meanings. An enhanced and broadly distributed N400 (starting at 300 ms) for incompatible conditions was observed, indicating that listeners can use online gestural information to disambiguate speech.

Previous results clearly suggest that co-speech gestures evoke semantic processing in the brain. However, an open question remains as to whether semantic processing engaged by gestures is qualitatively similar to the one evoked by linguistic material, such as words. To address this question, Ozyurek et al. (2007) presented subjects with auditory sentences in which a critical word was accompanied by videos of hand gesturing. In turn, the word, gesture, or both could be semantically anomalous with the preceding context. The results showed that incongruent conditions, either for words or gestures, produced a frontally distributed N400 (peaking at 480 ms) that had a similar amplitude, latency, and scalp distribution. Similar to the findings of Kelly et al. (2004), early differences were also observed (N1-P2). According to the authors, these results clearly demonstrate that the understanding of an utterance causes our brain to integrate semantic information conveyed through verbal and non-verbal modalities.

Surprisingly, mathematics is another domain where N400 effects have been observed. While previous studies have reported an “arithmetic N400 effect” during the processing of incongruous mental calculation problems (Niedeggen et al., 1999; Galfano et al., 2004), Lim et al. (2009) recently found an N400 effect for words describing mathematical functions (e.g., “diverging” and “quadratic”) primed by movies depicting incongruent gestures of these functions. In line with the findings of Ozyurek et al., 2007, this study reported that the topography, latency, and amplitude for the mathematical gestures are comparable to those observed for words.

Finally, another set of studies investigated the role of gestural information in the understanding of non-literal language. Cornejo et al. (2009) presented videos of an actor uttering metaphorical expressions and producing hand gestures that were either congruent or not with the metaphorical meaning of those expressions. It was found that gesture incongruity with a metaphorical expression elicited an N400 response (during 350–650 ms window) at the left-frontal region, followed by a LPC in right posterior sites. Although this study is the first to explore the integration of gestures and figurative language, it has certain limitations due to the absence of contrasts between literal and metaphorical stimuli. Consequently, Ibanez et al. (2011a,b) extended these findings by comparing literal and metaphorical expressions paired with congruent or incongruent gestures. In line with Cornejo et al. (2009) results, they found an N400 effect for incongruent gestures paired with metaphorical expressions over the left anterior regions, followed by a LPC for congruent gestures. According to the authors, these results suggest that the metaphorical meaning is available at the early stages of semantic processing and is highly sensitive to context.

Moreover, the contextual integration of speech and co-speech gestures is influenced by the semantic proficiency of a given language. In a another study, Ibanez et al. (2010) replicated previous findings and further showed that high level second language speakers are able to process and integrate gestures and linguistic expressions in a similar manner to native speakers.

Taken together, these findings are comparable, in terms of the anterior distribution of the effect, to those observed for everyday actions (Wu and Coulson, 2005; Kelly et al., 2007; Ozyurek et al., 2007; Cornejo et al., 2009; Ibanez et al., 2010, 2011a,b). Again, early anticipatory effects are reported in this domain with a bias over the left hemisphere in some cases (Cornejo et al., 2009; Ibanez et al., 2010, 2011a,b). In summary, available evidence suggests that gestures and words are processed by the brain in a qualitatively similar manner, supporting the coupling of language and sensorimotor systems during meaning construction.

## The N400 effects for motor events embedded in semantic contexts

Finally, a third domain of growing interest, including the coupling between language and action systems, is the one offered by the engagement of semantic processing during preparation and execution of goal-directed actions. In fact, much of our daily behavior is guided by “action semantics” (Van Elk et al., 2009), that is, a particular type of knowledge about how to interact with objects in an appropriate manner (e.g., how our body can interact with a cup in order to prepare coffee). This ability can sometimes be undervalued because it does not necessarily require further awareness. However, neurocognitive impairments, such as ideational apraxia (a dysfunction characterized by the loss of conceptual knowledge about the function of tools), highlights the crucial role that semantics plays for action execution (Van Elk et al., 2008).

Although there are not many studies on motor events using the N400 as an index of semantic processing, recent data have shed some insight into the temporal dynamics underlying semantics for action. For example, Van Elk et al. (2008) investigated the role of semantic knowledge in action planning. Participants were required to prepare meaningful or meaningless actions (e.g., bring a cup toward the mouth or toward the eye, respectively) and made a semantic categorization response before executing the corresponding action. In addition, words that were presented could be either congruent or incongruent with respect to the action-goal that subjects had to prepare. The results showed that the preparation of meaningful actions elicited a larger N400 for incongruent words (e.g., the word “eye” when they have to bring a cup to their mouth) compared to congruent words (e.g., the word “mouth”). This effect was observed during a 424 to 488 ms window and the distribution was found to be maximal over the fronto-central electrodes. Interestingly, no difference was found in the N400 amplitude when subjects had to prepare meaningless actions. According to the authors, these findings indicate that semantic knowledge is only activated during the preparation of meaningful actions or, more specifically, when people intend to use objects in a meaningful way.

In another study, Aravena et al. (2010) investigated the bidirectional impact of language and motor processes by using a slight modification of the action–sentence compatibility effect (ACE) paradigm (Glenberg and Kaschak, 2002). The ACE can be defined as a longer reaction time (RT) in the action-sentence incompatible conditions than in the compatible conditions. During the task, participants had to listen to sentences describing an action that could involve an open hand (e.g., applauding), a closed hand (e.g., hammering), or no manual action (e.g., visiting). Afterwards, subjects were required to press a button (either with an open or closed hand) to indicate the full comprehension of the sentence. Incompatible conditions (e.g., an open hand action sentence followed by a closed hand button response) gave rise to a central N400, suggesting that motor processes interfere with sentence comprehension. In addition, the modulation of motor potentials (MP) revealed a semantic facilitation of the motor response during congruent conditions. According to Aravena et al. (2010), reported data can be understood in terms of a dynamic co-operation model in which linguistic and motor-related activity can be dissociated but can also operate together in the context of a larger neural network.

**Figure 4:**
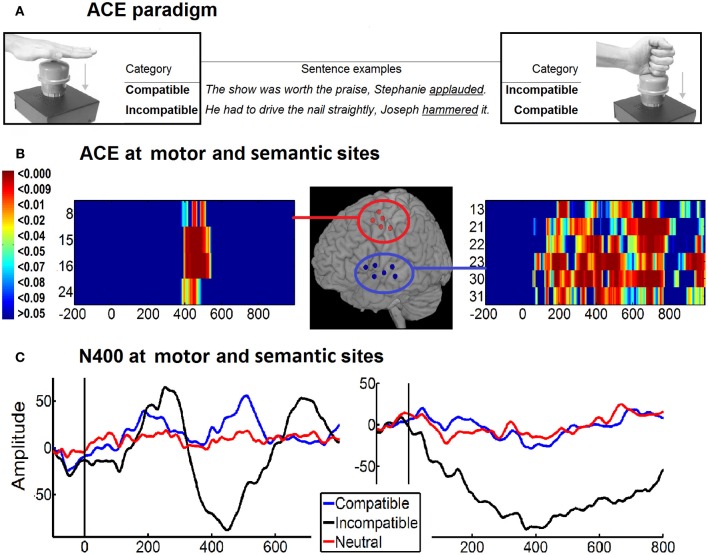
**ECoG of action-sentence compatibility effect. (A)** Example of ACE paradigm and stimuli. Both top corners shown participants hands position during the task (open at left and closed at right). In the center are two examples of the sentences according to the hand-shape of the action (open hand or closed hand sentences). The combined hand position used to depress the response button and the sentence content determines the type of category: compatible or incompatible. Final target verbs are underlined. **(B)** Motor and semantic areas producing an ACE. Normalized position of the electrodes showing a significant ACE (compatibility effect: incompatible minus compatible differences) at IFG, STG, and MTG (semantic-related areas, Blue circle) and the PM and M1 (motor-related areas, red circle). The time-probability charts show electrodes significant effects at N400 windows [M-N400 localized in premotor/motor (right side) and at temporal areas (left side)]. **(C)** Intracranial ERPs of Motor N400 (390–500 ms) and temporal N400 (250–700 ms) for compatible, incompatible and neutral categories. Modified with authorization from Cortex (Ibáñez et al., 2012a).

Similarly, in a recent study Ibanez et al. (2012b) measured the ACE effect in language (in the N400 window) and motor areas (in the MP window) with direct electrocorticography (ECoG) recordings in epileptic patients (Figure 4). They found that motor preparation affected language processing and vice versa. In the first case, the incongruent trials elicited a more negative amplitude in the signal than the congruent trials in movement-related areas such as premotor and M1. In the second one, language related-areas (STG, MTG, and left IFG) elicited a more negative response in the incongruent condition than in the congruent one. According to the authors, these results clearly support the bidirectionality hypothesis (Aravena et al., 2010) which claims that action-language comprehension and motor processes share neural resources that co-operate mutually during semantic processing.

In short, these studies suggest that the relationship between language and action is bidirectional and that it is present during action execution and motor planning. It is important to note that two of the three studies reviewed in this section (Van Elk et al., 2008; Ibáñez et al., 2012a) reported that N400 frontal distribution is observed for action-related material while the other one did not (Aravena et al., 2010). Thus, further work is needed in this particular domain to clarify this issue.

## The anatomical sources of the N400 for words and the N400 for actions

Using different techniques, several attempts have been made in order to disentangle the neural basis of the N400. Since these efforts have mainly been directed toward the semantic processing of words in either visual or auditory modalities, the generators of the N400 elicited by meaningful actions still remain unknown.

### The anatomical sources of the classic N400 for words

Converging evidence derived from evoked magnetic fields (Helenius et al., 1998, 2002; Halgren et al., 2002; Maess et al., 2006; Service et al., 2007; Vistoli et al., 2011), event-related optical signals (Tse et al., 2007), and intracranial recording studies (Halgren et al., 1994a,b; Guillem et al., 1995, 1999; McCarthy et al., 1995; Nobre and McCarthy, 1995) indicates that the classic N400 effect for words reflects the coordinated activity of multiple cortical areas, including the superior (STG) and the middle temporal gyri (MTG), superior temporal sulcus (STS), the anterior medial temporal lobe (AMTL), and inferior parietal sites (AG). Interestingly, some studies have also reported a widespread activation in frontal areas. For example, Halgren et al. (2002) found that differential activation to incongruous words in a semantic context began in temporal sites (Wernicke's area and antero-ventral temporal lobe) at 250 ms after word onset. However, following 300 ms, prefrontal areas (e.g., IFG and DLPFC) became increasingly activated. While these activations were observed in the left hemisphere, the right one got significantly involved after 370 ms. Similarly, Maess et al. (2006) reported the involvement of the left IFG and a bilateral activation in temporal areas (STG, ITG) for anomalous sentence endings. This bilateral activation observed in both studies is consistent with a growing body of data suggesting an important but lesser contribution of the right hemisphere in meaning processing (Hagoort et al., 2009). Furthermore, this activation becomes more bilateral as the semantic complexity of the information being processed increases (Federmeier et al., 2008).

Using similar experimental manipulations to those used to elicit the N400 effect (e.g., comparing semantically congruent/incongruent sentence endings), neuroimaging studies have also contributed to a better understanding of the neural basis of semantic processing (for a review of these fMRI studies see Lau et al., 2008). Overall, the most commonly reported areas across studies are the left STG/MTG, the IFG and the AG. Converging evidence for an involvement of these areas is also found in the MEG, intracranial, and fMRI studies reviewed in this section, suggesting that they play a key role in the generation of the N400 effect.

### The anatomical similarity of the N400 for actions and the classic N400

Previous source findings hold mainly for words but only partially for action meaning. One testable hypothesis is that, in the latter case, motor and premotor regions, such as domain-specific areas, would also be recruited during the processing of action-related information. Based on the scalp-recorded and the intracranial activity, three ERP studies have recently attempted to determine the neural sources of the action-elicited N400 effect. In the first study (Proverbio et al., 2010), source reconstruction using swLORETA (Palmero-Soler et al., 2007) located the generators of this effect in the left inferior, left middle, and right superior temporal regions (BA 20, 21) parietal areas (AG, BA 39), frontopolar regions (BA 10), bilateral premotor areas (BA 6), right posterior cingulate cortex, and extrastriate cortex. In the second study (Van Elk et al., 2010a) the stronger N400 effect for meaningful actions compared to meaningless actions was localized in the left premotor area (BA 6). Finally, the third one localized the effect in the STG, the MTG, the left IFG (pars opercularis and pars triangularis), and the premotor and M1 areas (Ibáñez et al., 2012a). Although limited and not conclusive, findings provided by these studies are in line with our previous assumption about the motor/premotor engagement during action meaning processing. In addition, it is important to note that an ERP study using verbal material about actions which have attempted to find the neural sources of the N400 effect have also reported the activation of motor and premotor cortical regions (see Van Elk et al., 2010b).

Convergent evidence coming from behavioral and ERPs studies of action priming shows an interplay between action-related and conceptual information (Helbig et al., 2006, 2010; Kiefer et al., 2011). In these studies, when source analysis is reported, generators for the fronto-central component within the sensory-motor systems and for the N400 within the anterior temporal lobe are observed.

Previous fMRI studies on action understanding that have used similar stimuli and/or experimental manipulations of those used for eliciting the action N400 represent a potential source of complementary evidence. For example, observing erroneous actions and meaningless movements lead to activations in premotor areas, with a main contribution of the left premotor cortex during the processing of object-related actions and a right contribution during the analysis of movements (Manthey et al., 2003). In addition, it has been reported that when we view meaningless movements, fronto-parietal regions of the perception action system are recruited (Hetu et al., 2011).

Observation of incorrect object-directed actions also activates, in a bilateral fashion, the IFG, premotor, temporal (STG, MTG, STS), and parietal regions (Newman-Norlund et al., 2010). Furthermore, daily actions performed in a compatible context generate significant activations in the left IFG and the superior part of the ventral premotor cortex (Wurm and Schubotz, 2012). Similar context effects have also been reported in motor/premotor areas and temporal regions (e.g., parahippocampal gyrus) in response to actions performed with inappropriate objects (pantomimes), taking place at incompatible contexts (Wurm et al., 2012).

In the speech and co-speech gestures domain, mismatching gestures in a language context lead to an increasing activation of premotor regions. Consistent with these findings, recent work on language and gesture processing (Willems et al., 2007; Holle et al., 2008; Dick et al., 2009; Hubbard et al., 2009; Kircher et al., 2009) also points to the engagement of temporal areas (STS, STG, MTG), inferior parietal (AG), IFG, and premotor regions in the interplay of action and language.

**Figure 5:**
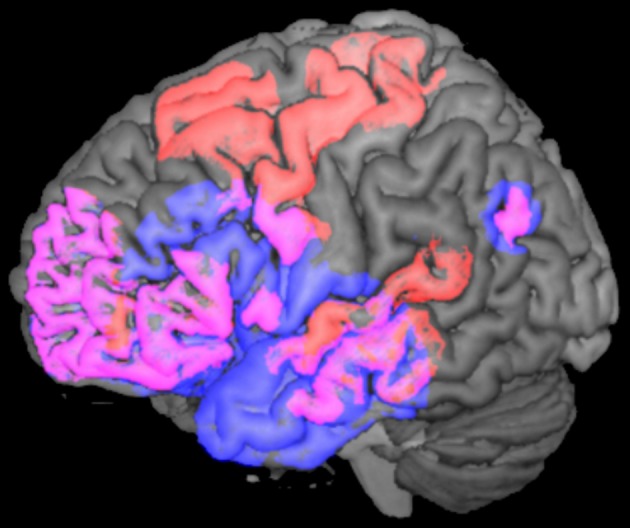
**N400 brain activations for words and actions.** Lateral view of the left hemisphere showing the N400 neural sources for words (in blue) and for actions (in red). The figure was computed using the MRIcron software (Rorden and Brett, 2000) and the *spherical regions* of *interest* (*ROIs*) (*5 mm*) displayed in the picture were taken from the MEG, fMRI, ERP, and intracranial studies reviewed in this article (please see Halgren et al., 2002; Proverbio et al., 2010; Ibáñez et al., 2012a). Please note that overlapping activations (in pink) in frontal, temporal, and parietal areas are common to both N400s, while motor and premotor regions are activated only during the processing of action-related material.

Taken together, convergent evidence derived from MEG, ERP, and fMRI studies supports the existence of a widely distributed semantic network, comprising a set of overlapping areas for both N400s in the frontal, temporal, and parietal lobes, with additional involvement of the motor and premotor regions in the particular case of action-related material (Please see Figure 5).

## Discussion

### Overall findings

The main purpose of this article was to offer a comprehensive characterization of the N400 for actions by reviewing current findings on this specific domain and to propose a functional neuroanatomical model that is able to integrate the action-related data to current knowledge about the classical N400 elicited by words.

As shown by the reviewed studies, the negative activity elicited by action-related anomalous stimuli begins early, approximately at 250–300 ms post-stimulus onset; perhaps reflecting the rapid access that realistic visual images have to semantic memory networks (West and Holcomb, 2002; Sitnikova et al., 2003, 2008; Mudrik et al., 2010). Nevertheless, other relevant literature, which also includes early components modulation without reporting the N400 (Hauk and Pulvermuller, 2004; Kiefer et al., 2007; Hauk et al., 2008), are out of the scope of this review. Note that in some N400 studies, even earlier modulations -in the 100 to 200 ms window- are observed when dynamic realistic visual images such as videos (Kelly et al., 2004, 2007) or static realistic images such as photographs (Proverbio and Riva, 2009; Proverbio et al., 2010) are used (see Figure 1). Accordingly, these particular temporal dynamics observed when real world features are presented could be reflecting a more direct and rapid mapping to sensorimotor representations.

In addition, the presence of a LPC following the N400 effect was reported in several studies (e.g., Sitnikova et al., 2003, 2008; Wu and Coulson, 2005; Cornejo et al., 2009; Ibanez et al., 2010, 2011a,b). This late effect is assumed to reflect accessing the knowledge of goal-related requirements about real-world actions (Sitnikova et al., 2008), a decision-making related process (Wu and Coulson, 2005), or a continued re-analysis of the inconsistent situation (Munte et al., 1998; Hurtado et al., 2009). Nevertheless, what the presence of this component suggests is that meaning is not computed at once, but rather it is something that emerges through time, with the N400 representing an important aspect of that emergent process, but not, certainly, the final state (Kutas and Federmeier, 2011).

No clear hemispheric dominance is observed across studies. While some studies report a bias over the left hemisphere (Cornejo et al., 2009; Ibanez et al., 2010, 2011a,b), others report that the N400 effect is more prominent over the right hemisphere (West and Holcomb, 2002; Reid and Striano, 2008). Thus, further research is needed to understand the lateralization profiles of different experimental designs and stimuli types.

Finally, the more anterior topographical localization often reported in N400 studies where non-verbal material is used, is also present. In consonance with neural source localization findings discussed in the previous section, this difference has led to the hypothesis that while both negativities could be reflecting similar functional operations instantiated by a common semantic network, these operations could be carried out in non-identical neuroanatomical substrates, with the coupling of motor/premotor regions in the particular case of actions. Although this hypothesis might seem obvious, the claim that meaning is grounded, wholly or in part, in systems for perception and action, is far from being trivial and is currently a debated topic in cognitive neuroscience.

### Language and sensorimotor processing: does the N400 for actions support a grounded view of meaning?

Classical linguistics theories (Collins and Loftus, 1975; Fodor, 1983; Masson and Borowsky, 1998) interpret meaning as the result of the combination of abstract, amodal symbols arbitrarily linked to entities in the real world. In this view, the sensorimotor information derived from our experiences with the world is completely detached from the conceptual knowledge that we have of it. One of the main difficulties derived from these theories, however, is the so-called grounding problem: if we want to know the meaning of an abstract symbol, the symbol has to be grounded in something other than more abstract symbols. The reason is simple: manipulation of abstract symbols merely produces more abstract symbols, not meaning (Glenberg and Robertson, 2000).

An alternative psycholinguistic approach, the embodied semantic theory, gained popularity in the last few years. One of the most radical and controversial claims in this field, suggests that language processing recruits a particular type of neurons that fires both during action execution and during action observation of the same/similar action: the mirror neurons (diPellegrino et al., 1992). In a strict sense, this theory predicts that mirror regions that are activated during action observation and action execution should also be activated during the comprehension of words referring to actions (Gallese and Lakoff, 2005; Pulvermuller et al., 2005; Gallese et al., 2007). Furthermore, these later semantic activations would be distributed in a somatotopically-arranged manner; with leg concepts (such as “kicking”) activating the homunculus leg area, mouth concepts (such as “eating”) activating the mouth area and so on.

The embodied framework has triggered intense discussions (Negri et al., 2007; Willems and Hagoort, 2007; Mahon and Caramazza, 2008; Toni et al., 2008; Hickok, 2009), and current neuroscientific research does not necessarily support its radical versions (Arevalo et al., 2012; Ibáñez et al., 2012a). Recent findings also suggest that the somatotopical activation pattern reported in many of these studies are not exact (Turella et al., 2009; Fernandino and Iacoboni, 2010) and that when the three conditions (observation, execution, and linguistic comprehension) are tested together in the same set of participants, activations elicited by action-associated linguistic stimuli do not match with the activations observed for execution and observation (Postle et al., 2008; de Zubicaray et al., 2010). In other words, “mirror areas” are not sufficient in explaining how our brain processes action meaning and the engagement of other cortical regions is clearly required (Brass et al., 2007).

Accordingly, more lenient versions predicting partially overlapping (but not identical) regions comprising a general motor-language network have been proposed. These interpretations come from studies reporting activity in regions outside the motor/premotor cortices such as the IFG, the temporal cortex, the cerebellum and the inferior/superior parietal lobule (Pobric and Hamilton, 2006; Gazzola and Keysers, 2009; de Zubicaray et al., 2010; Kemmerer and Gonzalez-Castillo, 2010). In consonance with these results, the source localization studies on the N400 for actions reviewed here report similar activations in the aforementioned regions, supporting a “grounded” approximation to meaning construction. Indeed, it has been suggested that the N400 component can be understood within an embodied framework (Chwilla et al., 2007, 2011; Collins et al., 2011; Hald et al., 2011). For instance, Chwilla et al. (2007) reported N400 modulations for novel senseless meanings compared to novel sensible meanings [e.g., “the boys searched for branches/bushes (sensible/senseless) with which they went drumming … ”]. While the first option makes sense, the second one does not. This is because the affordances of bushes do not mesh with the actions required to drum. Moreover, this study shows that participants can establish novel meanings not stored in memory, challenging abstract symbol theories that can only access meaning by consulting stored symbolic knowledge.

Hald et al. (2011) found a frontal N400 response, modulated by the modality switch effect. This effect occurs when a first statement -describing an event grounded in one modality- is followed by a second one in a different modality. For instance, “The cellar is dark” (visual property) followed by “A mitten is soft” (tactile property). The modality of the previous statement serves as a context and guides predictions. Accordingly, the statement “The cellar is … ” preceded by a tactile context leads to a weaker activation of “dark” than when the preceding context is visual. This is because that, guided by the tactile context, the system is looking for a tactile property of the “cellar,” and this will lead to a modality switch negativity. According to the authors these ERP results support an embodied and predictive view of language comprehension. Similarly, Collins et al. (2011) also found that the modality switching effect was associated with increased N400 amplitudes, supporting the claim that perception and action systems help subserve the representation of concepts.

Taken together, these studies are in line with the more lenient versions of the embodied approach and support a “grounded” view of the N400, in the sense that the retrieval of sensory and motor information clearly modulates meaning-related processes indexed by this component. In other words, comprehension has a contextual and situated nature and semantics are grounded in prior experiences with the world.

We believe in a *bidirectional cooperative* approach in which language and sensorimotor activity can be dissociated (Mahon and Caramazza, 2008), but can also operate together, during meaning construction, in the context of a larger network (Aravena et al., 2010). According to this view, meaning constitutes a polymodal, context-dependent, and constructive representation instantiated by the aforementioned distributed network (Amoruso et al., 2011, 2012; Ibanez and Manes, 2012).

### Context integration: the N400 action model

The presentation of incongruent vs. congruent verbal and non-verbal stimuli in different formats, such as environmental sounds, drawings, static, and dynamic pictures, all give rise to a similar N400 effect. Moreover, this effect has been reported at several levels of processing, including semantic, syntactic (Weber and Lavric, 2008; Zhou et al., 2010; Zhang et al., 2011; Morgan-Short et al., 2012), and phonological-orthographical levels (Deacon et al., 2004; Meng et al., 2008). In addition, other complex processes, such as metaphor (Cornejo et al., 2009; Ibanez et al., 2010, 2011a,b), irony (Cornejo et al., 2007), and joke comprehension (Coulson and Wu, 2005), have been shown to modulate the N400 amplitude. In brief, current electrophysiological evidence suggests that the N400 can be elicited by a wide range of stimuli as long as they are potentially meaningful (Kutas and Federmeier, 2011).

One common characteristic reported across studies is that as the target stimulus becomes more expected/congruent with the context, the N400 amplitude is reduced when compared with unexpected/incongruent conditions. This general finding, observed for stimuli across modality, suggests that when the previous context builds up meaning the processing of upcoming information that fits with the current context is facilitated. These effects, sometimes known as “cloze-probability” and “semantic incongruity,” respectively, remain stable across stimulus-modality.

Note, however, that unexpected sentence endings have been shown to elicit larger N400 responses, even when endings were semantically congruent (Kutas and Hillyard, 1984). Therefore, it is likely that this component reflects a more general process, than semantic processing *per se*, in which meaning is shaped by predictions that we create based on current contextual cues and previous experiences. For example, observing someone hammering a nail into a wall with a rolling pin is “weird” to our brain; however, it would not be strange if we knew that this person does not have a hammer and they managed to find an alternative solution in order to perform the action. In other words, meaningful actions depend on the circumstances, and a given stimulus can be classified as congruent or incongruent depending on the scenario and the predictions that we make from it.

Current research has shown that the brain is constantly benefiting from context by making predictions about future events (Bar, 2004, 2009). Predictive theories in the domain of perception and action suggest that our brains are good at reducing discrepancies between expectations and current experience. For instance, in the action field, predictive motor theories (Wolpert and Flanagan, 2001; Wolpert et al., 2003; Kilner et al., 2007a,b) assume that analogs models are used to generate predicted sensory consequences of executed actions and to inferred motor commands from observed actions. For example, the predictive coding account (Kilner et al., 2007a,b; Kilner, 2011) argues that intentions can be derived through action observation by the generation of an internal model that minimizes the prediction error at different levels of a cortical hierarchy. More specifically, by observing a person performing a specific action, we are able to predict their motor commands and, given these commands, we are able to predict their kinematics, by mapping this information into our own action system. When comparing this information on the multiple levels of the hierarchical model, a prediction error is generated. By minimizing this error at all the levels of action representation, we can infer the most likely cause of an observed action. In neuroanatomical terms, this model is thought of as a double pathway model where action understanding is achieved through interactions between a ventral pathway and a dorsal one (Kilner, 2011). While the ventral pathway links the MTG with the anterior IFG, the dorsal one refers to the action-observation network (AON), including the ventral premotor cortex, the inferior parietal lobule and the STS. The proposal here is that a representation of more abstract features (e.g., the intention and goal of an observed action) is generated by the ventral pathway, through a process of semantic retrieval and selection. This result in the encoding of the representation of the most probable action required to achieve the most probable goal. Once this goal is estimated, then a prediction of the sensory consequences of this action (a more concrete representation of the action) can be generated by the dorsal pathway.

In the perceptual field (Bar, 2004, 2009), object recognition is thought to be mediated by cognitive structures (memory scripts) that integrate information about the identity of the objects that tend to co-occur in a given context with previously learned information about their possible relationships. These structures are thought of as a set of expectations about what is more probable to see or not to see in a given context, enabling us to make predictions and accurately disambiguate incoming information. In this model, frontal areas are involved in updating current contextual information and integrating it with semantic associations stored in temporal regions (e.g., parahippocampal and retrosplenial cortex).

In consonance with the aforementioned accounts, we propose a model for the N400 for actions where frontal areas (e.g., IFG) would update ongoing contextual information in working memory and integrate it with learned target-context associations stored in temporal regions (MTG, STS) in order to get the specific significance of an action event (Amoruso et al., 2011, 2012; Ibanez and Manes, 2012). In addition, the inferior parietal lobe, as a cross-modal area, would mediate the integration of sensory, motor, and conceptual information (Seghier, 2013). Indeed, strategic connections between frontal, temporal, sensorimotor, and parietal regions involved in intentional (Waszak et al., 2012) and conceptual (Opitz, 2010) binding-related processes, such as linking actions to their predicted effects, have been proposed. Based on this account, the N400 can be seen as a neural marker that indexes the integration of current contextual cues. This later process involves: (1) prediction-related activity (frontal regions) and (2) integration with previous experiences (temporal and parietal regions). In addition, the retrieval of modality-specific information (e.g., motor-related information) facilitates the overall process as it becomes well-illustrated in forward models about action.

When we observe another person performing a given action such as grasping a glass of water, we are able to accurately anticipate the future course of the observed action. In other words, current contextual information and previous similar experiences enable as to predict incoming steps and shape meaning construction. These expectations are triggered at different levels, with top–down (e.g., expectations about the intention or the action goal) and bottom–up (kinematics and motor commands) information working together in a mutually constraining manner. Based on this view, our model provides an empirically testable set of hypotheses regarding contextual-based prediction and action meaning comprehension in N400 paradigms. For instance, during tasks using realistic visual images about actions, we expect to observe the engagement of the aforementioned fronto-temporo-parietal network working in concert with motor/premotor areas. In other words, we expect that the semantic processing involved in the N400 effect for action-related material would trigger a sensorimotor resonance in the observer. This prediction is partially confirmed by studies showing that the observation of actions that can be directly mapped onto the observer's motor system report a significant activation of premotor areas (see Van Elk et al., 2008). In temporal terms, we expect that ERP modulations would be observed from its earliest stages, perhaps due to the direct sensorimotor mapping elicited by realistic stimuli. In fact, this is the case in most of the reviewed N400 studies using ecological material (e.g., videos) about everyday actions. Thus, if “grounding” information such as kinematics, body movements, and interactions with artifacts or body/body parts is crucially required by the task (as in most of the designs used in N400 studies for actions) we expect that activity in motor/premotor areas will be enhanced and rapidly observed. In addition, we expect that during the integration of language-related stimuli (e.g., utterances) and action material (e.g., gestures) fronto-temporo-parietal regions as well as motor/premotor regions would be equally activated and maybe a delay in the N400 latency could be reported.

However, it remains an open question if this predictive account for actions could be extended to those tasks where the processing of the incongruence only relies on the use of language-material. While contextual cues clearly serve to pre-activate features of likely upcoming words (e.g., Ibanez et al., 2006, 2011a,b), such that the processing of unexpected stimuli that share semantic features with predicted items is facilitated (Kutas and Federmeier, 2011), it is unclear if a similar predictive error triggered during verbal semantic processing at different levels (e.g., words, sentences, pieces of discourse) can be explained in terms of forwards models. Future studies would benefit the validation and development of the proposed model by defining more detailed and testable predictions including the specific measures of the aforementioned processes.

In particular, our notion of context-dependent construction of meaning based on frontotemporal circuits resembles the view laid out by other colleagues (Kiefer and Pulvermuller, 2012). They suggest that concepts are flexible, distributed and modality-specific sensory and action representations, which depend on previous experience. Kiefer and Pulvermüller also argue that conceptual information proper is stored in sensory and motor areas whereas the anterior temporal lobe serves as a convergence zone for binding the distributed modality-specific representations. In addition, meaning does not necessarily depend only on actions, but also on sensory information from different modalities such as visual form features, motion, sound (Simmons et al., 2007; Hoenig et al., 2008; Kiefer et al., 2008, 2012). This model resembles our bidirectional coupling between motor and language areas. But they differ in the emphasis on modality-specific sensory and action representations and in the somatotopic representations. Strong claims of modality-specific and somatotopic representations have been challenged and recently criticized (see a work summarizing several sources of evidence: Cardona et al., 2013). Moreover, the distributed and extended source of N400 does not fit adequately with a model of somatotopic representations. Our model predicts a coupling, without interpretations about explicit representation coming from discrete areas. Meaning represents an emergent property of such motor-language coupling itself. Thus, in our model meaning is an emergent property of the fronto-temporal network and not only of modality-specific representations.

Recent accounts have proposed the existence, in the anterior temporal lobe (ATL), of a mechanism supporting the interactive activation of semantic representations across modalities (Patterson et al., 2007). According to this position, sensorimotor and language aspects of conceptual knowledge are necessary but not sufficient to build up meaning and an amodal hub region which makes generalizations is required. However, this proposal, mainly derived from anatomo-clinical observations in patients with semantic impairments, is far from being consistent (see Gainotti, 2011). Although many temporal areas are involved in the generation of the action-related N400, the anterior parts of the temporal lobe are not reported when experimental paradigms use current actions or action observation (e.g., Proverbio et al., 2010; Van Elk et al., 2010a,b; Ibáñez et al., 2012a). In fact, the involvement of this cortical area is often seen in N400 tasks requiring only lexical representations (Halgren et al., 2002), suggesting that it might support basic combinatorial operations underling sentence processing (Dronkers et al., 2004; Lau et al., 2008) and syntactic aspects (Noppeney and Price, 2004). In the particular case of the N400 for actions, when determining the incongruence of a given stimulus clearly relays more on a sensorimotor resonance or the re-enactment (Barsalou et al., 2003) of perceptual and action-related states in order to get the meaning of an event, the role of the ATL would be an auxiliary one. Accordingly, its involvement is not expected in these later cases (as supported by source localizations studies on the N400 for actions reviewed in this paper), but it would be indeed expected when the processing or disambiguation of the incongruent incoming information requires more “abstract” operations -and this is the case (see N400 studies on word processing reviewed by Lau et al., 2008).

In brief, action N400 supports a fronto-temporo-parietal network (Gainotti, 2011) in which motor and semantic representations would operate together during comprehension of complex situations, predicting effects of semantic processing on the motor system and vice versa. In this view, we avoid predictions derived from radical embodiment (e.g., somatotopic activations) and we only take advantage of the proposal that sensorimotor “grounded” information derived from real-world experiences are necessary during the comprehension of perceived or produced events. Thus, the activation of this network would be modulated depending on stimulus type properties (indexing cortical related activations), previous experiences and learning effects (temporal regions), and current contextual predictions and expectations (IFG and other frontal regions).

## Conclusions and future directions

In conclusion, evidence summarized in this selective review suggests that, at a semantic level, action meaning and language meaning lead to qualitative similar N400 modulations. In the current review, we focused on N400 for actions, and did not include early ERP effects or a deeper discussion about meaning and neuroscience, which would be an important topic for future research.

We have proposed that this semantic process indexed by the N400 is accomplished by a fronto-temporo-parietal network in which meaning construction is shaped by predictions derived from contextual ongoing information and previous knowledge. By this means, we suggest that predictive and semantic-related processing are core aspects of what this component is actually indexing.

While we believe that meaning is a situated, pluralistic and multimodal phenomenon that goes beyond action and language *per se* and that both negativities are, at a general level, functionally equivalent, many questions await further answers. For example, although the activation of motor and premotor regions in action comprehension could partially explain the frontal pattern activation, and the temporal dynamics involved in this specific process (e.g., accessing the contextual network depending on stimulus type) still need to be elucidated. In other words, it is not clear if motor and premotor areas become directly activated by incoming action related-stimuli or if they are later recruited by the fronto-temporo-parietal network when conceptual processing has already occurred. In addition, further studies should specify the anatomical localization of the N400 effect for actions. Indeed, there is little evidence about the action N400 generators and, although it supports the engagement of temporal, frontal, and motor/premotor regions in action comprehension, further experimentation is clearly required to complement current results.

### Conflict of interest statement

The authors declare that the research was conducted in the absence of any commercial or financial relationships that could be construed as a potential conflict of interest.
